# Netherton syndrome presenting with generalized erythroderma in a preterm neonate: Diagnostic and therapeutic challenges

**DOI:** 10.1016/j.jdcr.2026.02.052

**Published:** 2026-09-03

**Authors:** Zahra Khodabakhshian, Naser Tayyebi Meibodi, Ahmad Reza Taheri

**Affiliations:** a Department of Dermatology, Faculty of Medicine, Mashhad University of Medical Sciences, Mashhad, Iran; b Department of Pathology, Imam Reza Hospital, Mashhad University of Medical Sciences, Mashhad, Iran; c Cutaneous Leishmaniasis Research Center, Faculty of Medicine, Mashhad University of Medical Sciences, Mashhad, Iran

**Keywords:** diagnose, generalized erythroderma, Netherton syndrome, preterm neonate

## Introduction

Netherton syndrome (NS) is a rare, severe autosomal recessive genodermatosis first described by Comèl in 1949 as ichthyosis linearis circumflexa and later by Netherton in 1958, who identified the characteristic hair shaft defect now known as trichorrhexis invaginata.^1^^,^^2^ NS results from biallelic pathogenic variants in the SPINK5 gene located on chromosome 5q32, encoding lymphoepithelial Kazal-type-related inhibitor.^2^^,^^3^ Clinically, NS typically presents in neonates or early infancy with generalized ichthyosiform erythroderma or scaly erythroderma.^1^, ^2^, ^3^ The hallmark hair abnormality, trichorrhexis invaginata (“bamboo hair”), may not always be evident at birth and often requires trichoscopy or light microscopy for diagnosis. Other hair shaft anomalies such as “golf tee” or “matchstick” hairs have also been described.^1^ Atopic manifestations are an integral part of NS and include severe eczematous lesions, food allergies, allergic rhinitis, asthma, and elevated serum immunoglobulin E levels. Immunological abnormalities, including hyper-eosinophilia and a Th17/Th2 skewed immune profile, contribute to the multisystemic nature of the disease.^2^, ^3^, ^4^

Therapeutic approaches have historically been limited to supportive care including intensive skin hydration, use of emollients, topical antibiotics, and infection control.^3^^,^^5^ However, recent advances have introduced systemic therapies such as intravenous immunoglobulin and biologic agents targeting IL-17A, IL-4Rα, and IL-12/23 pathways. Early mortality remains a major concern, especially in the first year of life. In the largest infant NS cohort to date, overall mortality was ∼9%, with ∼50% experiencing hypernatremic dehydration and ∼66% underweight at presentation. These data highlight the high early-life vulnerability in NS despite supportive care.^6^ The absence of a collodion membrane at birth can be a helpful differentiating feature compared to other congenital ichthyoses.^2^

Here, we report the case of a 21-day-old preterm neonate who presented with severe generalized erythroderma, confirmed by trichoscopic and histopathologic findings to have NS. Despite supportive interventions, the disease course in this patient was complicated by sepsis, and she ultimately expired during hospitalization.

## Case report

### Patient information

A 21-day-old female neonate was referred to the Dermatology Clinic at Imam Reza Hospital for evaluation and management of generalized erythroderma. She was born at 34 + 0 weeks gestation (late preterm) to consanguineous parents (first cousins) and was the third child in the family, following two healthy older brothers. There was no history of a collodion membrane at birth, as confirmed by the attending pediatrician. Maternal history was negative for any underlying medical conditions or medication use during pregnancy. A detailed family history revealed no evidence of dermatologic conditions such as ichthyosis, psoriasis, atopic dermatitis, or allergic diseases among first- or second-degree relatives.

### Clinical findings

On physical examination, the neonate presented with generalized erythroderma, characterized by extensive erythema, scaling, and crusting involving the entire body surface. Prominent periorbital edema and facial swelling were evident (Fig 1), reflecting the severity of the inflammatory response. Examination of the trunk showed widespread scaling with thick, plate-like areas of desquamation (Fig 2). Although the classic “double-edged” scaling pattern of ichthyosis linearis circumflexa was not distinctly visualized in the available image, the clinical morphology was suggestive of this pattern. Involvement of the extremities was also observed, with marked erythema and scaling most prominent over the lower limbs. The flexural areas showed enhanced desquamation and erythema, contributing to the overall characteristic appearance (Fig 3). To further investigate the diagnosis, trichoscopic examination of the eyebrow hair was performed, revealing trichorrhexis invaginata—a ball-and-socket deformity commonly referred to as “bamboo hair” (Fig 4), which is considered pathognomonic for NS.

**Fig 1:**
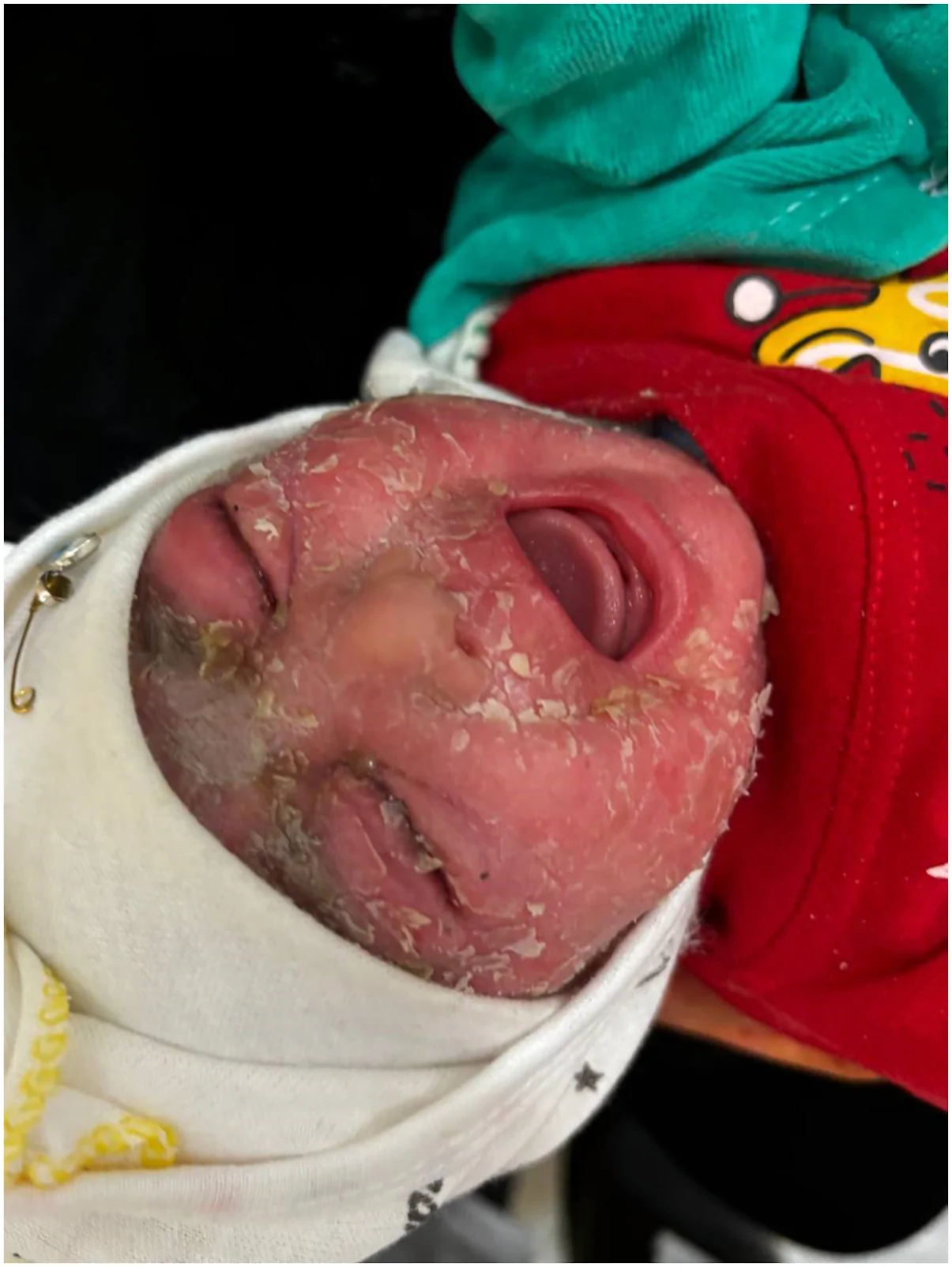
Prominent periorbital edema and facial swelling.

**Fig 2:**
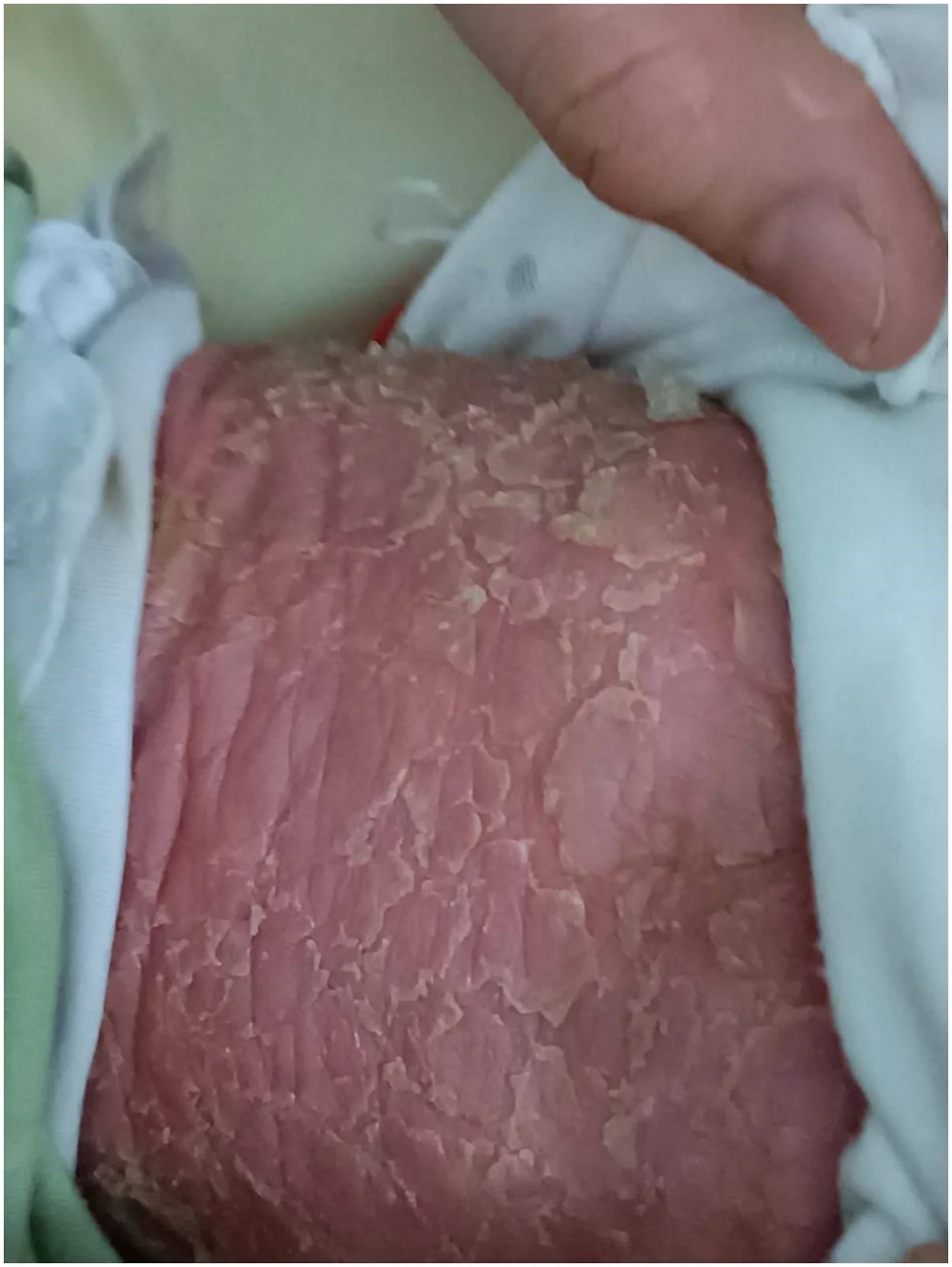
Trunk skin showing scales with a distinctive double-edged appearance.

**Fig 3:**
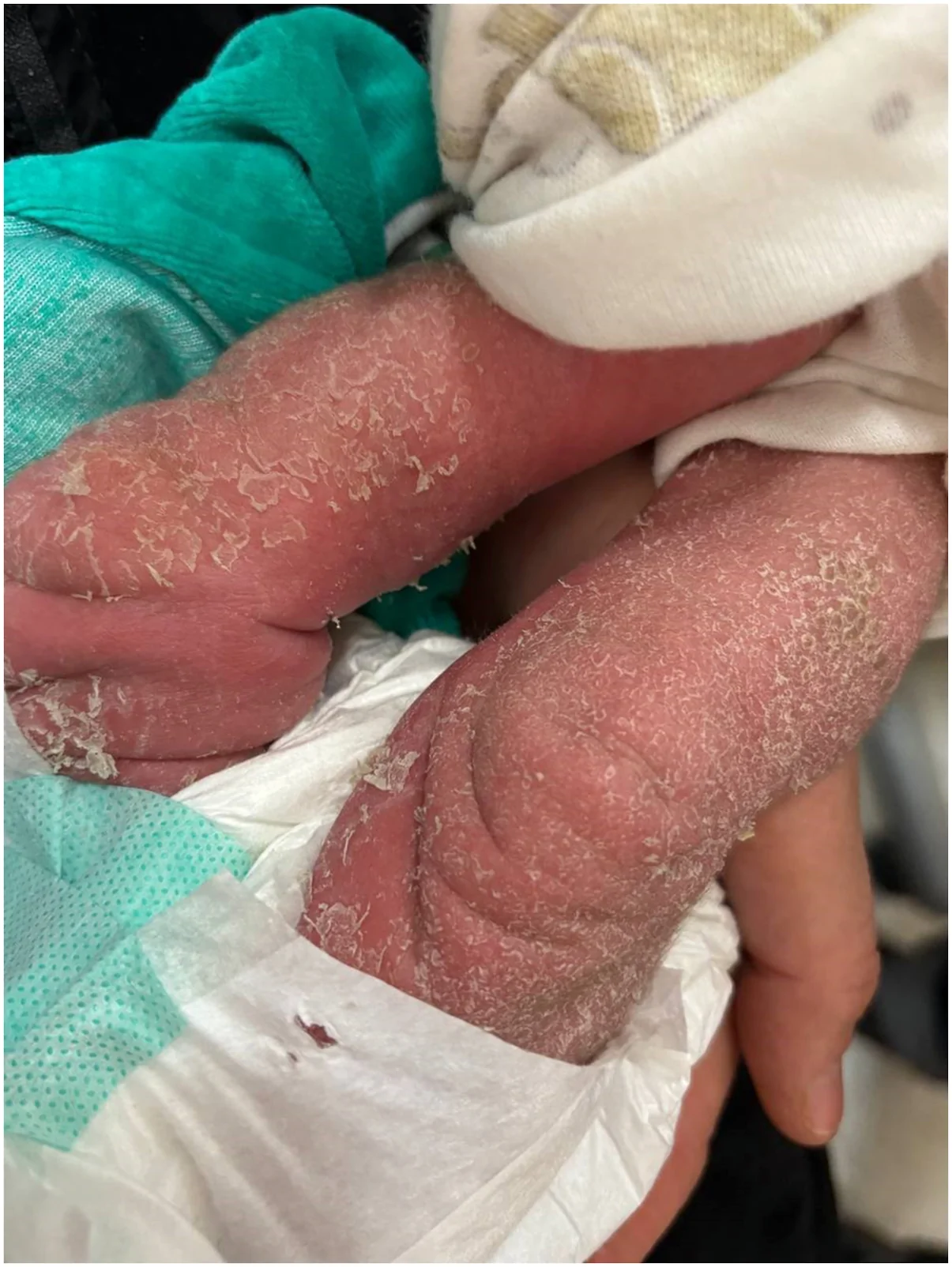
Scaling and erythema involving the lower extremities, with accentuation in flexural areas.

**Fig 4:**
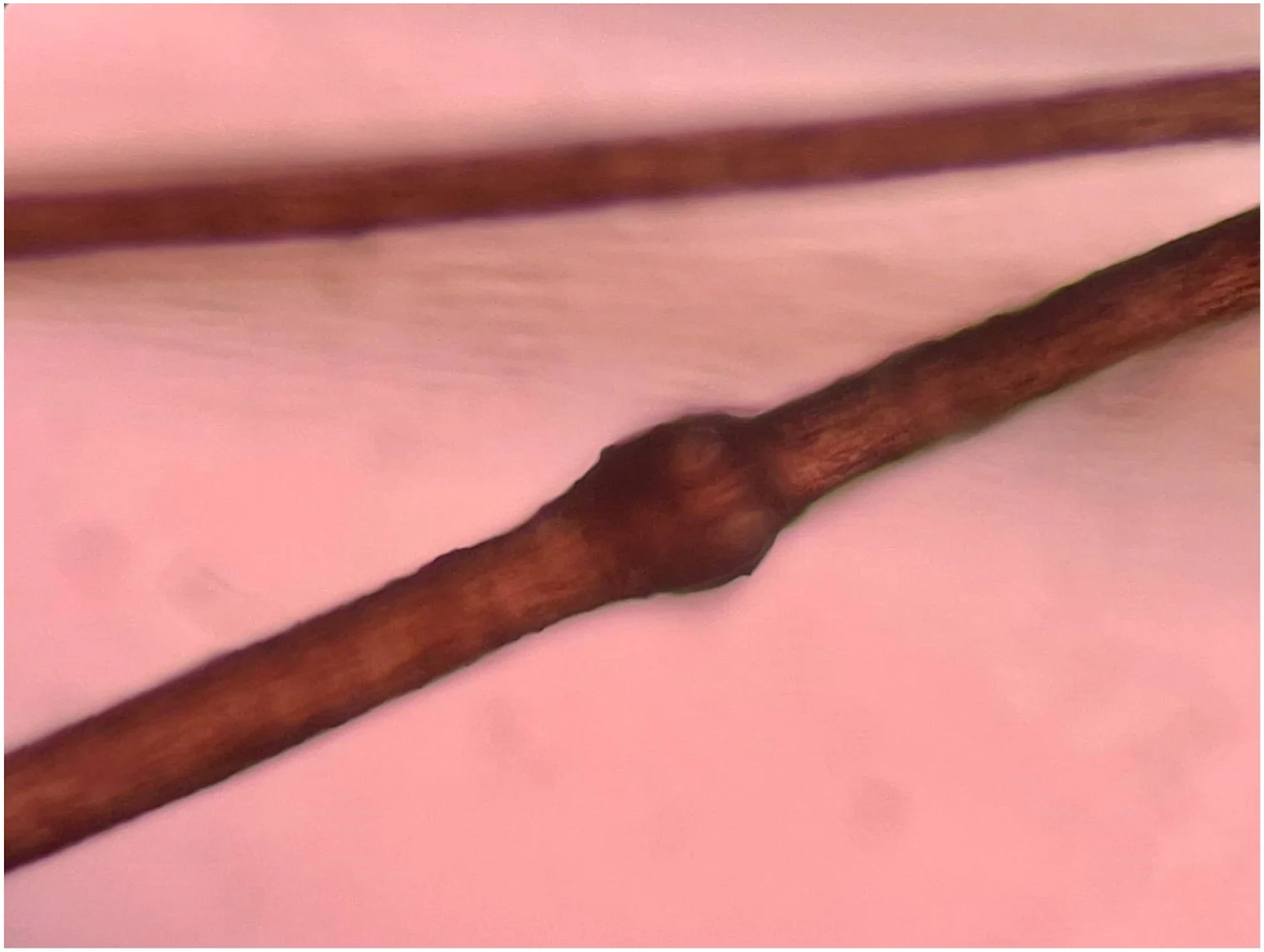
Abnormal hair shaft (eyelash) in Netherton syndrome. Trichorrhexis invaginata with its ball-and-socket appearance.

### Diagnostic assessment

Both a skin biopsy and trichoscopic examination of the eyebrow hairs were performed. Histopathological analysis of the skin biopsy revealed parakeratotic hyperkeratosis, acanthosis, and spongiosis, along with mild perivascular chronic inflammation in the superficial dermis (Figs 5 and 6). Although these features were non-specific, they were consistent with findings observed in ichthyosiform dermatoses and supported the clinical suspicion of NS. The trichoscopic examination demonstrated the presence of trichorrhexis invaginata—commonly referred to as “bamboo hair”—characterized by a ball-and-socket appearance of the hair shaft (Fig 4), which is pathognomonic for NS. Based on the clinical presentation, supportive histopathology, and definitive trichoscopic findings, a diagnosis of NS was established.

**Fig 5:**
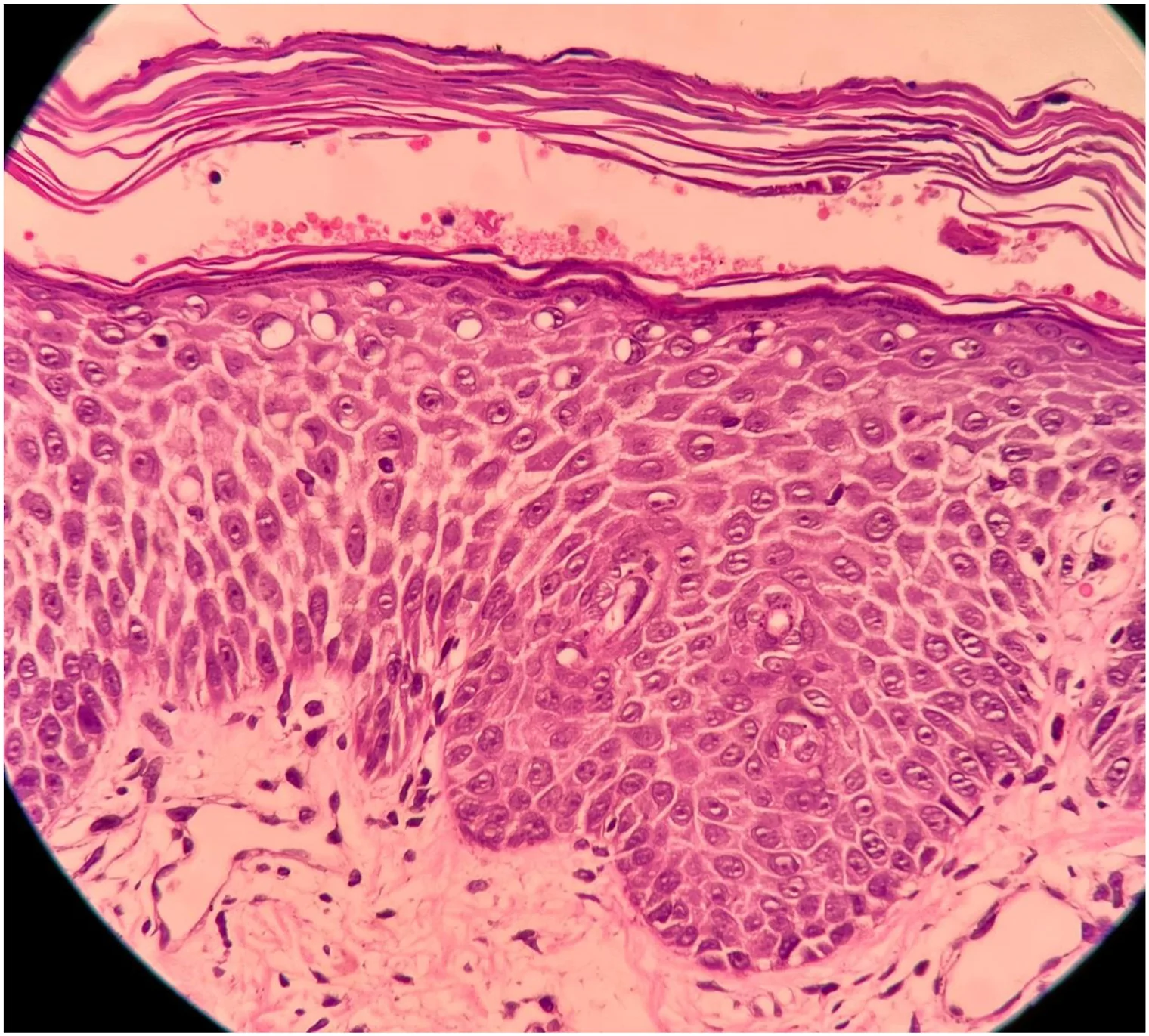
Histopathologic image of skin biopsy (H&E stain, original magnification × 10 and × 40). The epidermis shows parakeratotic hyperkeratosis, acanthosis, and spongiosis. A mild chronic inflammatory infiltrate is observed around superficial dermal vascular plexus.

**Fig 6:**
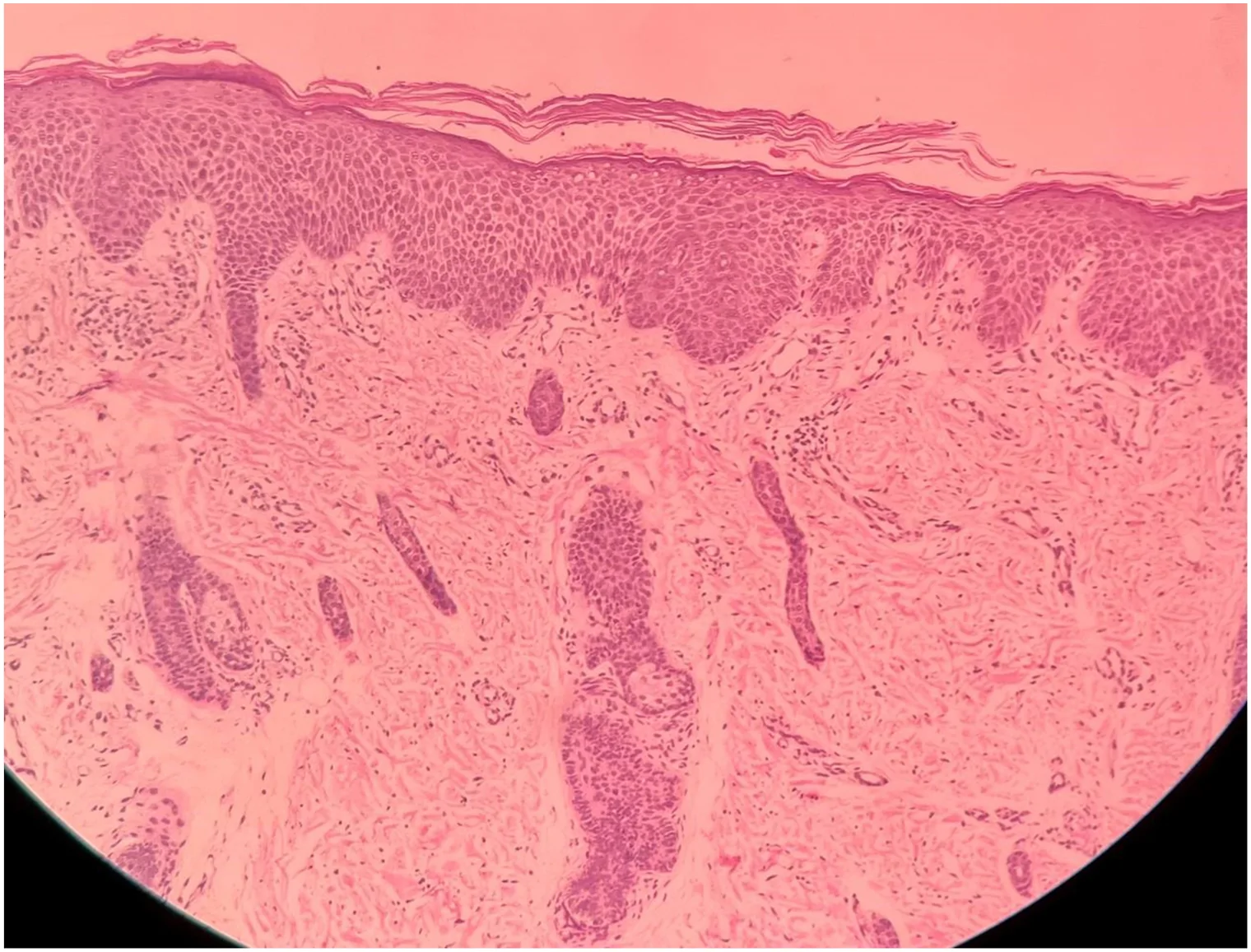
Histopathologic image of skin biopsy (H&E stain, original magnification × 10 and × 40). Magnification 10× H&E staining. Epidermis with parakeratotic hyperkeratosis, acanthosis, and spongiosis Around the superficial vascular network, mild chronic edema.

### Therapeutic intervention

Adequate hydration and maintenance of ambient room temperature were emphasized to mitigate further skin barrier disruption and reduce the risk of systemic complications. The patient was advised to wear soft, breathable cotton clothing to minimize mechanical irritation. Regular application of emollients and mild keratolytic agents was initiated to improve skin hydration and promote gentle desquamation. Topical antibiotics were also prescribed to help prevent secondary bacterial infections. In parallel, the patient was referred to a pediatric allergist-immunologist for further evaluation, including measurement of serum immunoglobulin E, eosinophil count, and consideration of genetic testing to identify pathogenic variants in the SPINK5 gene.

### Outcome and follow-up

During the course of further immunologic evaluation, the neonate developed signs of systemic infection, presenting with fever and pneumonia. Despite transfer to the ICU and initiation of intensive supportive care, the patient deteriorated rapidly and ultimately succumbed to sepsis. This unfortunate outcome underscores the severe morbidity and high mortality risks associated with NS in the neonatal period, particularly when complicated by systemic infections.

## Discussion

Our patient, a 21-day-old preterm neonate with generalized erythroderma, periorbital edema, and characteristic hair shaft abnormalities confirmed by trichoscopy, fits the typical early presentation of NS. Despite early supportive interventions, the disease course was complicated by pneumonia, sepsis, and ultimately fatal outcome, emphasizing the high neonatal morbidity and mortality associated with NS.^3^^,^^5^

Differential diagnosis of neonatal erythroderma is broad and includes congenital ichthyoses, Omenn syndrome, and severe atopic dermatitis.^1^^,^^2^ The absence of a collodion membrane at birth, combined with the double-edged scaling pattern and the trichoscopic finding of trichorrhexis invaginata, provided important diagnostic clues. Hair abnormalities, although pathognomonic, may not always be present at birth, necessitating repeated examinations.^1^^,^^2^

The immune defects contribute to the susceptibility to recurrent and severe infections,^3^^,^^7^ as seen in our patient who developed pneumonia and sepsis. The Frontiers in Oncology case report by Skoczen et al (2020) highlighted, for the first time, an association between NS and hematologic malignancy (acute lymphoblastic leukemia), suggesting that while NS is not classically categorized as a cancer predisposition syndrome, chronic immune dysregulation and impaired skin barrier might increase susceptibility to malignancies.^7^

Phototherapy, particularly narrowband UVB, has also been used with some success in selected cases.^3^ However, due to the patient's age and clinical instability, these interventions were not considered in the present case. Systemic interventions, such as intravenous immunoglobulin therapy, have shown benefits in select cases by enhancing immune function and reducing infection rates.^5^^,^^7^ Recent advancements propose the use of biologic therapies targeting inflammatory pathways, such as IL-17 and IL-4Rα inhibitors (eg, secukinumab, dupilumab), offering hope for improved disease control.^4^^,^^5^ However, these treatments are not yet standardized, especially in neonates, and their long-term safety profiles remain under investigation.

Importantly, emerging genetic studies suggest that certain SPINK5 mutations may correlate with more severe phenotypes, especially those leading to early truncation of lymphoepithelial Kazal-type-related inhibitor.^7^ NS should be considered in neonates presenting with generalized erythroderma, particularly in the context of consanguineous parentage and the presence of characteristic hair shaft abnormalities. Early clinical suspicion, supported by histopathologic and trichoscopic findings, is essential for timely diagnosis and management. Despite advances in supportive care, neonates with NS remain at high risk for severe complications, including hypernatremic dehydration, systemic infections, and sepsis. This case underscores the critical importance of early recognition, multidisciplinary management, aggressive infection surveillance, and close monitoring to improve outcomes in affected infants.

## Conflicts of interest

None disclosed.
